# Supplementary material for: Preparation of Bio-Foam Material from Steam-Exploded Corn Straw by In Situ Esterification Modification
Source: Polymers (Basel). 2023 May 8;15(9):2222. doi: 10.3390/polym15092222 (PMC10180570; doi:10.3390/polym15092222)
Supplement: Supplementary file 1 [file polymers-15-02222-s001.zip › polymers-2298325-supplementary.pdf]

Supplementary Materials

1. Preparation and Testing

Prior to the modification of straw, we also explored the esterification conditions of pure cellulose and lignin. The possibility of esterification of pure cellulose and lignin using a VA cross-linking reagent was determined.

1.1. Esterification of Cellulose

Firstly, 2.025 g of cotton pulp cellulose containing 0.0125 mol of dehydrated glucose unit (AGU) and 50 mL of DMSO were fully intensively mixed in a metal bath at 60 °C for 10 minutes; after that, 2.5 mL of 10 mol/L NaOH solution was added and stirred for another 5 minutes. Then, the mixture was reacted in an oil bath at 110 °C for 20 min with the addition of VA (the molar ratio of AGU to VA was 1:6) where the cellulose was fully activated. At the end of the procedure, the reaction was stopped by adding 200 mL of alcohol. Finally, the sample was washed with deionized water and ethanol until the filtrate was neutral and then it was dried in the oven at 60 °C.

Table S1. The orthogonal experimental design for the esterification modification of the steam-exploded corn straw.

Factor	level 1	level 2	level 3	level 4
A: Temperature (°C)	90	100	110	120
B: Time (min)	10	15	20	25
C: Amount of VA (mol)	0.04	0.06	0.08	0.10

1.2. Esterification of Lignin

In this process, 1 g of dealkaline lignin was weighed and dispersed in 40 mL of DMSO solvent, which was fully intensively mixed in the metal bath at 60 °C for 10 minutes; after that, 2.5 mL of 10 mol/L NaOH solution was added and stirred for another 5 minutes. Then, the mixture was reacted in an oil bath at 110 °C for 20 min with the addition of VA (the molar ratio of AGU to VA was 1:6) where the cellulose was fully activated. At the end of the procedure, the reaction was stopped by adding 200 mL of alcohol. Finally, the sample was washed with deionized water and ethanol until the filtrate was neutral, and then it was dried in the oven at 60 °C.

1.3. Esterification of Steam-exploded Corn Straw

The orthogonal experiment is shown in Table S2. The detailed pretreatment conditions of temperature, time, and the amount of esterification reagent (VA) on the reaction efficiency were studied.

Table S2. Summary of specific reaction conditions and DS results of esterification.

Number	Temperature(°C)	Time(min)	Amount of VA (mol)	DS (mmol/g)
s1	90	20	0.06	4.36
s2	90	10	0.04	3.83
s3	90	15	0.10	4.63
s4	90	25	0.08	4.85
s5	100	20	0.10	4.48
s6	100	15	0.06	4.17
s7	100	10	0.08	4.43
s8	100	25	0.04	3.83
s9	110	10	0.10	4.36
s10	110	15	0.04	3.45
s11	110	20	0.08	4.70

Number	Temperature(℃)	Time(min)	Amount of VA (mol)	DS (mmol/g)
s12	110	25	0.06	4.08
s13	120	25	0.10	4.70
s14	120	20	0.04	4.41
s15	120	15	0.08	4.22
s16	120	10	0.06	4.65

1.4. The Simulation Analysis Method with Finite Element

COMSOL software was used to simulate the thermal insulation performance of Lig-HCCP-SECs-4.5 foam at 60 ℃ and 100 ℃. The simulation parameters were obtained according to the tests of thermal conductivity and true density. The specific parameters are shown in the following Table S3.

Table S3. Modeling preferences of simulation analysis method of thermal insulation performance.

Parameters	Thermal conductivity （W/（m·K））	Heat capacity （J/（g·K））	Density （g/cm³）	Porosity（%）
Value	0.028	1.89	0.028	90.3

2. Results and discussion

2.1. Morphological Characterization of corn straw

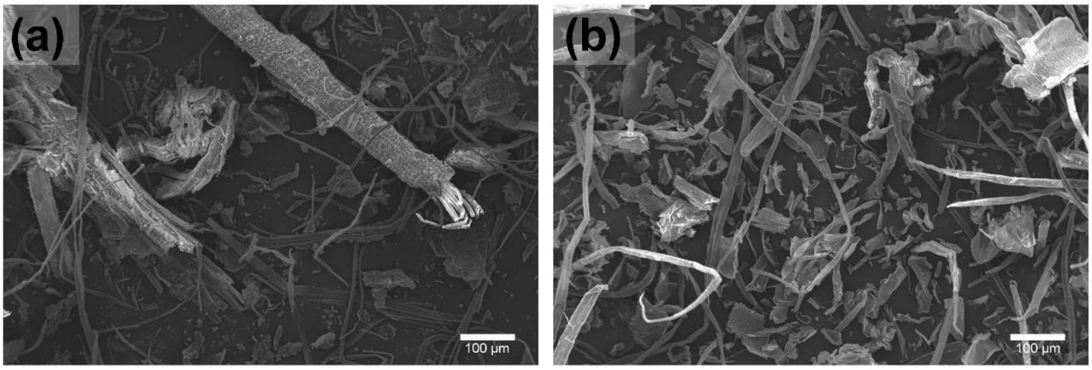

Figure S1. SEM images of raw corn straw (a); steam-exploded corn straw (b).

It can be seen from Figure S1a and Figure S1b, after the explosion treatment, that the internal fiber bundles were separated and crushed.

2.2. The Chemical Structure Characterization

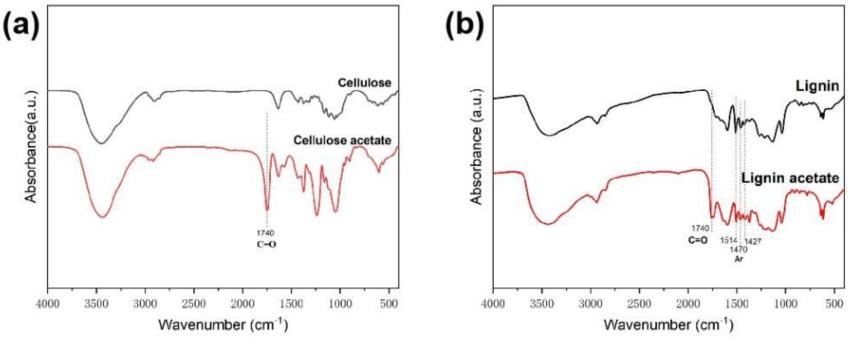

Figure S2. FT-IR spectra of pure cellulose **(a)** and lignin **(b)** before and after esterification.

The FTIR spectra of pure cellulose and lignin before and after esterification is shown in Figure S2. The absorption peak at 1740 cm^{-1} belongs to the C=O stretching vibration of acetyl groups, indicating that both had been successfully esterified. The possibility of esterification of pure cellulose and lignin using VA crosslinking reagent was determined.

The ^{13}C NMR spectra of esterified cellulose and lignin were detected and are illustrated in Figure S3.

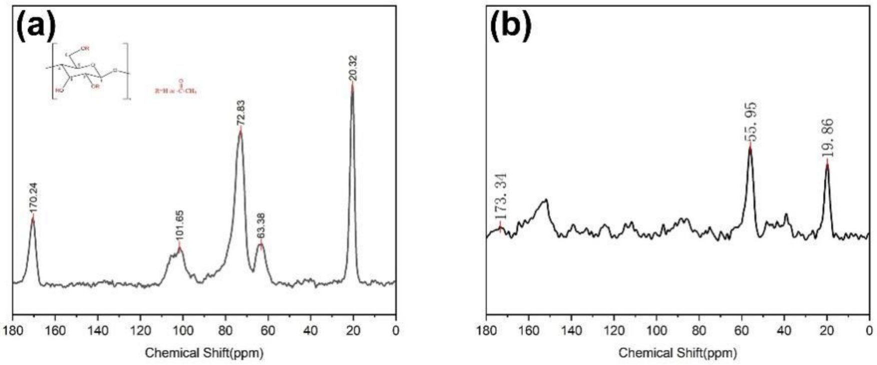

Figure S3. The ^{13}C NMR spectra of esterified cellulose **(a)** and lignin **(b).**

All the characteristic peaks were visible. As shown in Figure S3a, the appearance of the peaks at 170.3, 169.4, and 169.0 (C=O at C6, C3, and C2 positions, respectively), the peak located at 62–103 ppm (carbons of the anhydroglucose ring), and the peak at 20 ppm (CH_3) showed the successful synthesis of esterified cellulose[54]. At the same time, as shown in Figure S3b, the characteristic peaks at 173 ppm (COOH), 55.95 ppm (CH_3O), and 19.86 ppm (CH_3 in CH_3COO) appeared in the spectra which also illustrated that the acetylated lignin had been prepared [55].

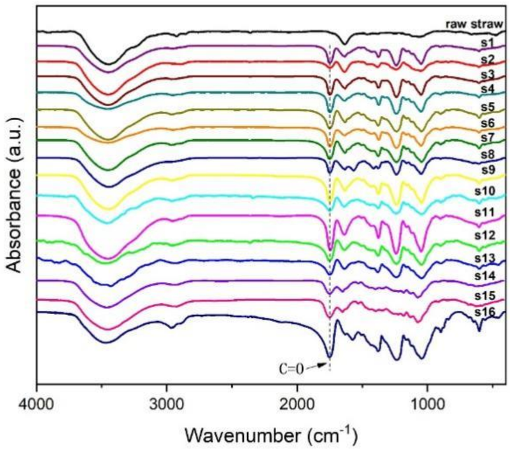

Figure S4. FT-IR spectra of raw straw and all esterified products.

For all the ECS_{-x} samples, the FTIR spectra are shown in Figure S4. The peak located at 1740 cm^{-1} was attributed to the stretching vibration of C=O, indicating the success of the esterification reaction. Noticeably, the greater the DS, the more intensive the peak at 1740 cm^{-1}, indicating that the content of C=O increased.
